# Does Melatonin Exert Its Effect on Ram Sperm Capacitation Through Nitric Oxide Synthase Regulation?

**DOI:** 10.3390/ijms21062093

**Published:** 2020-03-18

**Authors:** Sara Miguel-Jiménez, Melissa Carvajal-Serna, Silvia Calvo, Adriana Casao, José Álvaro Cebrián-Pérez, Teresa Muiño-Blanco, Rosaura Pérez-Pe

**Affiliations:** Department of Biochemistry and Molecular and Cell Biology, Faculty of Veterinary Sciences, Institute of Environmental Sciences of Aragón (IUCA), University of Zaragoza, Miguel Servet 177. 50013 Zaragoza, Spain; 652325@unizar.es (S.M.-J.); mcarvajals@unal.edu.co (M.C.-S.); silvianumber4@gmail.com (S.C.); adriana@unizar.es (A.C.); pcebrian@unizar.es (J.Á.C.-P.); muino@unizar.es (T.M.-B.)

**Keywords:** ram spermatozoa, capacitation, nitric oxide, melatonin

## Abstract

Nitric oxide (NO·), synthesized from L-arginine by nitric oxide synthase (NOS), is involved in sperm functionality. NOS isoforms have been detected in spermatozoa from different species, and an increment in NOS activity during capacitation has been reported. This work aims to determine the presence and localization of NOS isoforms in ram spermatozoa and analyse their possible changes during in vitro capacitation. Likewise, we investigated the effect of melatonin on the expression and localization of NOS and NO· levels in capacitated ram spermatozoa. Western blot analysis revealed protein bands associated with neuronal NOS (nNOS) and epithelial NOS (eNOS) but not with inducible NOS (iNOS). However, the three isoforms were detected by indirect immunofluorescence (IFI), and their immunotypes varied over in vitro capacitation with cAMP-elevating agents. NO· levels (evaluated by DAF-2-DA/PI staining) increased after in vitro capacitation, and the presence of L-arginine in the capacitating medium raised NO· production and enhanced the acrosome reaction. Incubation in capacitating conditions with a high-cAMP medium with melatonin modified the NOS distribution evaluated by IFI, but no differences in Western blotting were observed. Melatonin did not alter NO· levels in capacitating conditions, so we could infer that its role in ram sperm capacitation would not be mediated through NO· metabolism.

## 1. Introduction

Nitric oxide (NO·) is a free radical involved in various physiological processes, acting as a signalling molecule. There has been growing interest in the role of NO· in sperm motility [1], viability [2], hyperactivation [3,4], capacitation [5,6,7], acrosome reaction [8,9] and zona pellucida-binding [10]. Moreover, an antiapoptotic role has been attributed to NO· in spermatozoa [11].

Nitric oxide synthase (NOS) is the enzyme responsible for NO· production through the conversion of L-arginine to L-citrulline. Three isoforms of this enzyme have been identified to date: type I or neuronal (nNOS), type II or inducible (iNOS) and type III or endothelial (eNOS). Types I and III are constitutive isoforms, and their activation is regulated by calcium and calmodulin [12], whereas type II is independent of calcium, although in mice, it binds calmodulin tightly [13]. Constitutive isoforms are responsible for physiological NO· levels (nM levels) in somatic cells, whereas inducible NOS is involved in the production of higher levels (µM levels) under intracellular or extracellular inflammatory stimuli [14,15,16]. 

All isoforms of this enzyme have been described in human [17,18,19], bovine [20,21], murine [22] and boar [9] spermatozoa, among others. Furthermore, it has been demonstrated that NOS activity increases during in vitro capacitation in human sperm [5]. However, to the best of our knowledge, there are no previous studies identifying any NOS isoforms in ram spermatozoa. 

In mammals, melatonin is secreted by the pineal gland in response to light stimulus, and, apart from other functions, it modulates the secretion and synthesis of gonadotropic hormones via the hypothalamic-pituitary axis [23]. Thus, melatonin regulates reproductive seasonality in some species, such as ovine. Apart from pineal melatonin, this molecule is synthetized in other tissues and organs, including those in the male reproductive tract, as we recently demonstrated in ram [24]. Melatonin is also present in reproductive fluids such as seminal plasma [25] or follicular fluid [26]. This could explain how melatonin affects ram sperm functionality directly, modulating processes such as capacitation and decreasing apoptosic markers and oxidative stress [27]. The antioxidant capacity of melatonin in ram spermatozoa could be the reason for its antiapoptotic action, since high levels of reactive oxygen species (ROS) lead to cell death [28,29,30,31]. The ROS include superoxide anion (O_2_-), hydrogen peroxide (H_2_O_2_), hydroxyl (OH) and nitric oxide (NO·) (also included in the reactive nitrogen species, RNS). All these molecules play an important role in sperm capacitation (reviewed by [32]).

Melatonin can modulate NOS activity and, consequently, the nitric oxide in somatic cells [33,34,35]. Du Plessis et al. [28] concluded that melatonin reduces NO· levels in human spermatozoa in vitro, but they were not able to determine whether the decrease in NOS activity might be the reason for this reduction.

The main objective of this study was to determine whether the effect of melatonin on ram sperm capacitation was mediated by an action on NO· metabolism. For this purpose, we first focused on the detection of nitric oxide synthase isoforms in ram spermatozoa (1). We then analyzed the changes in the protein expression profile and localization of NOS isoforms during in vitro capacitation (2). Moreover, we aimed to determine the involvement of nitric oxide in ram sperm capacitation, using a NO· precursor (L-arginine) and a NOS competitive inhibitor (NG-nitro-L-arginine methyl ester, L-NAME) (3). Finally, we investigated how melatonin affected the expression and localization of NOS and NO· levels in capacitated ram spermatozoa (4). 

## 2. Results 

### 2.1. Nitric Oxide Synthase Immunodetection and Immunolocalization

**Figure 1:**
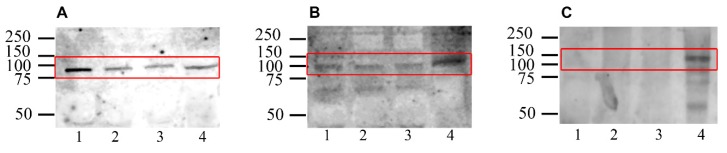
Western blot analysis of the presence of nitric oxide synthase isoforms eNOS (**A**), nNOS (**B**) and iNOS (**C**) in swim-up selected (lane 1) and in vitro capacitated without (control, lane 2) or with cAMP-elevating agents (Cap-C, lane 3) ram sperm protein extracts. Positive controls: rat lung extract (RLE, lane 4 in A) for eNOS, mouse brain extract (MBE, lane 4 in B) for nNOS and mouse macrophage activated with IFNɣ/LPS lysate (LPS-L, lane 4 in C) for iNOS.

Western blot analysis identified protein bands with a molecular weight corresponding to eNOS (~100 kDa) and nNOS (~120 kDa) in swim-up and in vitro capacitated samples (control and capacited control, Cap-C), but no bands associated with iNOS were detected (Figure 1). The molecular weights of these bands matched those found in the positive controls. 

**Figure 2:**
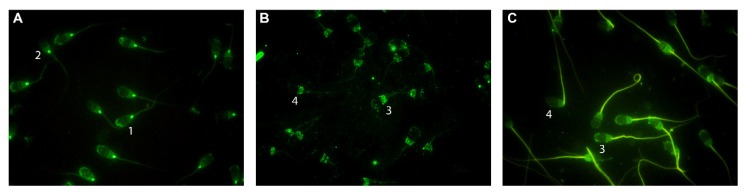
Indirect immunofluorescence (IFI) localization of nitric oxide synthase isoforms eNOS (**A**), nNOS (**B**) and iNOS (**C**) evaluated by fluorescence microscopy in ram spermatozoa. Two immunotypes can be seen in each isoform: labeling in postacrosomal region + neck + apical edge (1) or postacrosomal region + neck (2) in eNOS (A), postacrosomal region + apical edge (3) or postacrosomal region (4) in nNOS (B) and iNOS (C). Original magnification ×1000.

Immunofluorescence analysis revealed the presence of the three isoforms in ram spermatozoa. Two different immunotypes for each isoform were distinguished. Immunotype 1 with labeling at the postacrosomal region, neck and apical edge, and immunotype 2 with labeled postacrosomal region and neck, were distinguished for eNOS (Figure 2A). Immunotype 3 with labeling at the postacrosomal region and the apical edge and immunotype 4 labeling the postacrosomal region were distinguished in nNOS (Figure 2B) and iNOS (Figure 2C). iNOS also showed brighter fluorescence in the flagellum in both immunotypes 3 and 4 (Figure 2C). 

### 2.2. Role of Nitric Oxide in Ram Sperm Capacitation

**Figure A1:**
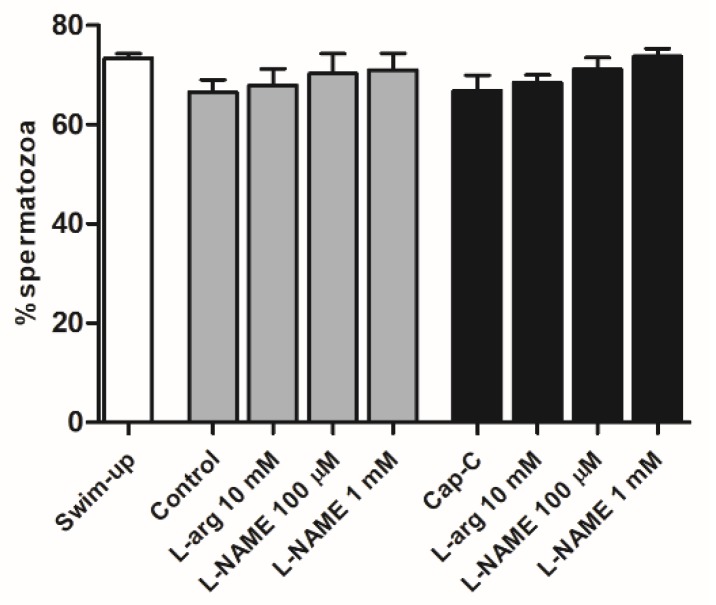
Percentage of viable spermatozoa before (swim-up, white bars) and after in vitro capacitation without (control, grey bars) and with cAMP-elevating agents (Cap-C, black bars) and with L-arginine (L-arg, 10 mM) or L-NAME (100 µM or 1 mM) (n = 7).

Once the presence of NOS in ram spermatozoa had been demonstrated, we evaluated the changes in the levels of its product, i.e., nitric oxide (NO·), after incubation in capacitating conditions. To further investigate the involvement of NO· in ram sperm capacitation, samples were incubated with a nitric oxide precursor, L-arginine at 10 mM, or with a NOS inhibitor, NG-nitro-L-arginine methyl ester (L-NAME) at 100 µM and 1 mM. The capacitation status together with motility and viability were evaluated. Viability, according to membrane integrity, was not compromised in any of the experimental conditions (Appendix A, Figure A1).

#### 2.2.1. Changes in Nitric Oxide Levels During Incubation in Capacitating Conditions

**Figure 3:**
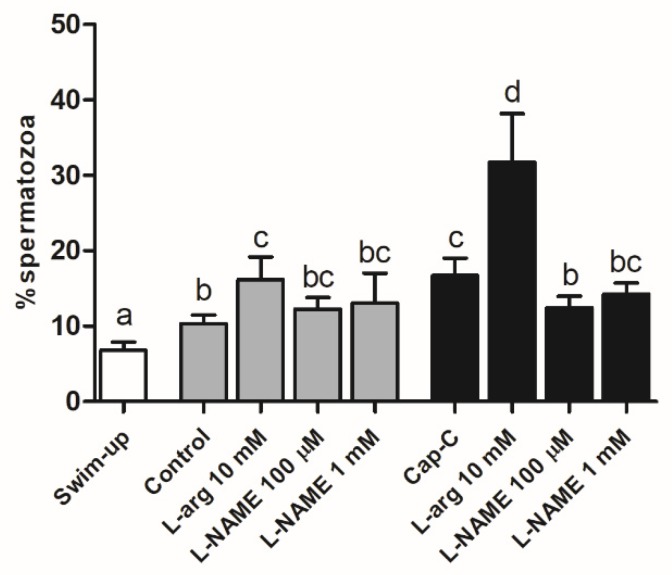
Percentage of live sperm with high nitric oxide levels (PI-/DAF+) assessed by flow cytometry before (swim-up, white bars) and after in vitro capacitation without (control, grey bars) and with cAMP-elevating agents (Cap-C, black bars) and with L-arginine (L-arg, 10 mM) or L-NAME (100 µM or 1 mM). Data are shown as mean ± S.E.M. (*n* = 6). Different letters indicate significant differences (*p* < 0.05).

Nitric oxide levels were assessed by flow cytometry, and the results are shown in Figure 3. The percentage of live sperm with high NO· levels (PI-/DAF+) significantly increased (*p* < 0.05) after in vitro capacitation without (control) and with cAMP-elevating agents (capacitated control, Cap-C). The addition of 10-mM L-arginine (L-arg) in both the control and Cap-C samples led to a significant increment (*p* < 0.05 and *p* < 0.0001, respectively) in the percentage of live spermatozoa with high NO· levels. Additionally, the NOS inhibitor, L-NAME, at 100 µM reduced (*p* < 0.05) the nitric oxide production in capacitated sperm (Cap-C), but no effect was appreciated in the control samples. 

#### 2.2.2. Influence of Nitric Oxide on Capacitation Status

**Figure 4:**
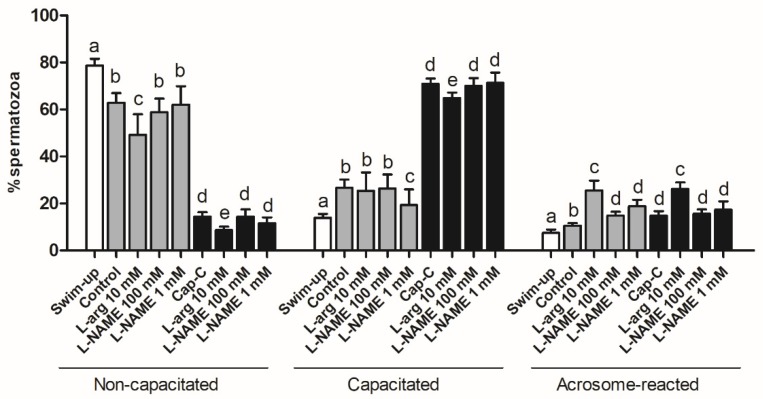
Assessment of capacitation status, evaluated by chlortetracycline(CTC), in ram spermatozoa before (swim-up, white bars) and after in vitro capacitation without (control, grey bars) and with cAMP-elevating agents (Cap-C, black bars) and with L-arginine (L-arg, 10 mM) or L-NAME (100 µM or 1 mM). Data of noncapacitated, capacitated and acrosome-reacted spermatozoa are mean percentages ± S.E.M (*n* = 8). Different letters within the same group indicate significant differences (*p* < 0.05).

The chlortetracycline (CTC) analysis revealed significant differences between samples (Figure 4). As expected, incubation in capacitating conditions produced a significant (*p* < 0.001) decrease in noncapacitated spermatozoa, concomitant with an increase in the percentage of capacitated and acrosome-reacted cells, which was much more marked in samples with c-AMP-elevating agents (Cap-C). The increase in NO· levels by the addition of L-arginine (Figure 3) gave rise to a significantly higher percentage of acrosome-reacted spermatozoa (Figure 4) in the control (25.5% ± 4.2% in control + L-arg vs. 10.5% ± 1.15% in control) and capacitated samples (26.13% ± 2.86% in Cap-C + L-arg vs. 14.75% ± 1.93% in Cap-C) (*p* < 0.001). Concomitantly, the percentage of noncapacitated (8.63% ± 1.48%) and capacitated (64.87% ± 2.31%) spermatozoa decreased significantly (*p* < 0.05) in Cap-C samples with L-arg in comparison with its own control, Cap-C (14.37% ± 1.91% noncapacitated and 70.88% ± 2.23% capacitated sperm). However, the slight reduction in NO· levels provoked by the addition of L-NAME (Figure 3) did not exert an effect on sperm samples during capacitation (Figure 4).

#### 2.2.3. Influence of Nitric Oxide on Sperm Motility

**Figure 5:**
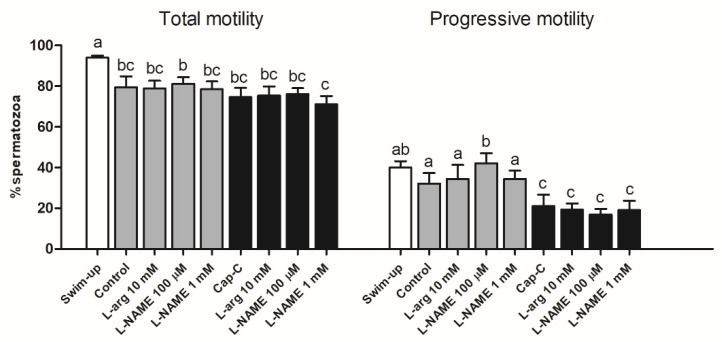
Percentage of total motile (left) and progressive (right) spermatozoa before (swim-up, white bars) and after in vitro capacitation without (control, grey bars) and with cAMP-elevating agents (Cap-C, black bars) and with L-arginine (L-arg, 10 mM) or L-NAME (100 µM or 1 mM). Data are shown as mean ± S.E.M. (*n* = 5). Different letters indicate significant differences (*p* < 0.05).

Total motility declined significantly after in vitro capacitation, but it was not affected by the increase or decrease of NO· levels when L-arginine or L-NAME were added, respectively (Figure 5). Progressive motility did not change in the control samples compared to the swim-up group, but L-NAME at 100 µM increased significantly (*p* < 0.05) the percentage of progressive motile spermatozoa (Figure 5). Samples capacitated in the presence of c-AMP-elevating agents (Cap-C) showed a significant reduction in progressive motility in comparison with the swim-up and control samples, but incubation with L-arginine or L-NAME in these conditions did not affect the progressive motility results.

### 2.3. Effect of Melatonin on the Nitric Oxide Metabolism

After confirming the presence of nitric oxide synthase in ram spermatozoa and the influence of nitric oxide on in vitro capacitation, the next step was to determine whether the effect of melatonin on ram sperm capacitation was mediated by the interaction with the nitric oxide metabolism.

**Figure 6:**
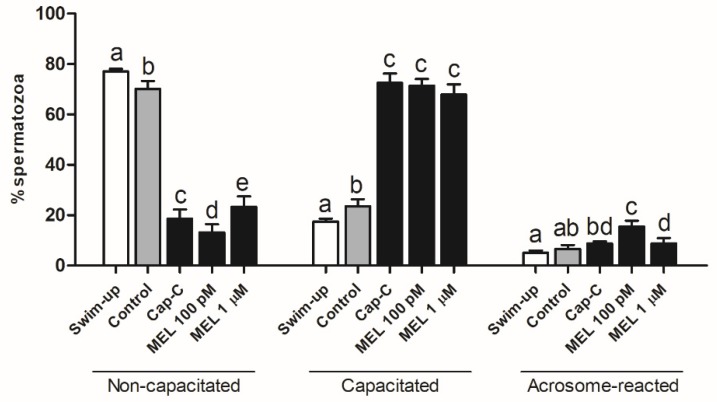
Assessment of capacitation status, evaluated by CTC, in ram spermatozoa before (swim-up, white bars) and after in vitro capacitation without (control, grey bars) and with cAMP-elevating agents (Cap-C, black bars) and with melatonin (MEL, 100 pM or 1 µM). Data of noncapacitated, capacitated and acrosome-reacted spermatozoa are mean percentages ± S.E.M (*n* = 7). Different letters within the same group indicate significant differences (*p* < 0.05).

CTC staining (Figure 6) showed that melatonin added to capacitated samples (Cap-C) caused a decrease in the percentage of noncapacitated spermatozoa at 100 pM and, in contrast, an increment with a 1-µM concentration (*p* < 0.05). The percentage of acrosome-reacted sperm was significantly higher in samples treated with 100-pM melatonin (15.5% ± 2.36% vs. 8.83% ± 0.79% in Cap-C).

#### 2.3.1. Effect of Melatonin on Nitric Oxide Synthase Levels and Immunolocation

**Figure 7:**
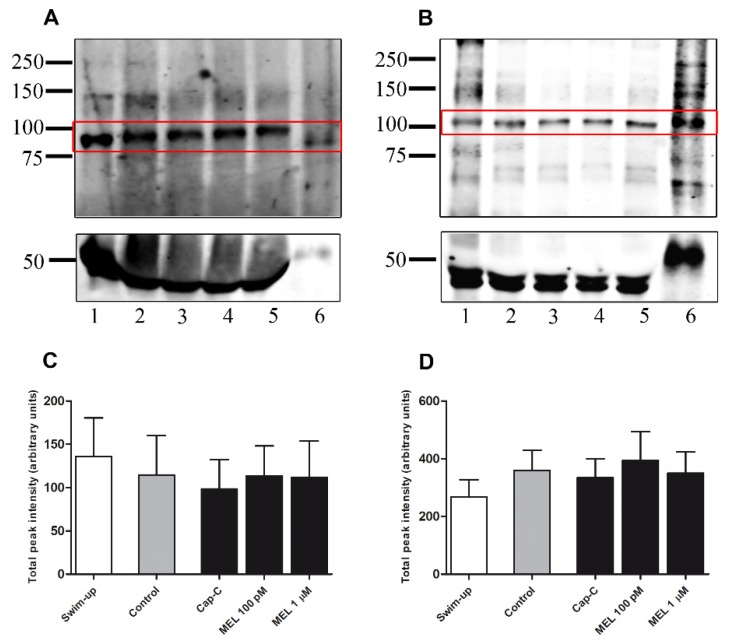
Western blot analysis of the presence of nitric oxide synthase isoforms eNOS (**A**) and nNOS (**B**) in swim-up selected (lane 1) and incubated 3 h in capacitating conditions without (control, lane 2) or with cAMP-elevating agents (Cap-C, lane 3) ram spermatozoa protein extracts. Effect of melatonin (MEL) 100 pM and 1 µM in capacitated samples with high cAMP (lanes 4 and 5). Positive controls: rat lung extract for eNOS (RLE, lane 6 in A) and mouse brain extract for nNOS (MBE, lane 6 in B). Densitometry quantification of eNOS (**C**) and nNOS (**D**) normalized to α-tubulin (loading control) analyzed by Western blot (*n* = 4).

In order to study whether the concentration-dependent effects of melatonin on ram sperm capacitation were mediated by acting on the nitric oxide synthase, we investigated changes in eNOS and nNOS levels by Western blot in capacitated (Cap-C) samples incubated with melatonin at 100 pM and 1 µM (Figure 7A,B). Changes in iNOS were not investigated, given that it was not detected by Western blot. After quantifying NOS bands for each isoform by densitometry, in relation to the α-tubulin loading control (~50kDa), no significant differences were found between samples (Figure 7C,D).

**Figure 8:**
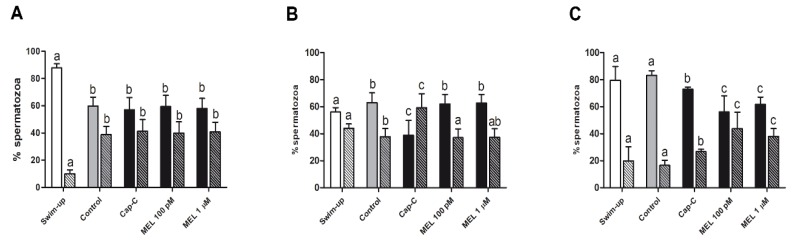
Percentages of the immunotypes for eNOS (**A**), nNOS (**B**) and iNOS (**C**) in swim-up selected (white bars) and in vitro capacitated ram spermatozoa without (control, grey bars) or with cAMP-elevating agents (Cap-C, black bars), and with melatonin (MEL) 100 pM and 1 µM. Plain bars represent immunotype 1 for eNOS (postacrosomal region + neck + apical edge) and immunotype 3 for iNOS and nNOS (postacrosomal region + apical edge), whereas striped bars represent immunotype 2 (postacrosomal region + apical edge) for eNOS and immunotype 4 (postacrosomal region) for iNOS and nNOS. Results are shown as mean ± SEM (*n* = 5). Different letters indicate statistical differences between treatments (*p* < 0.05).

However, when we analysed the proportion of the different immunotypes for each NOS isoform, changes associated with in vitro capacitation could be observed (Figure 8). The addition of cAMP-elevating agents (Cap-C sample) led to a decrease (*p* < 0.05) in the percentage of spermatozoa showing labeling on the sperm apical edge in the three isoforms (immunotype 1 for eNOS and 3 for nNOS and iNOS). This reduction became more pronounced in the presence of melatonin (100 pM and 1 µM) for iNOS (56.0% ± 12% and 61.67% ± 12%, respectively, vs. 72.83% ± 1.59% in the Cap-C sample; *p* < 0.05; Figure 8C). In contrast, for nNOS, immunotype 3 became predominant again when capacitated samples were incubated with melatonin (62.67% ± 6.3% and 62.00% ± 7.01% for 100 pM and 1 µM, respectively; *p* < 0.05; Figure 8B). No changes in eNOS immunotypes were observed in the presence of melatonin (Figure 8A).

#### 2.3.2. Effect of Melatonin on Nitric Oxide Levels

**Figure 9:**
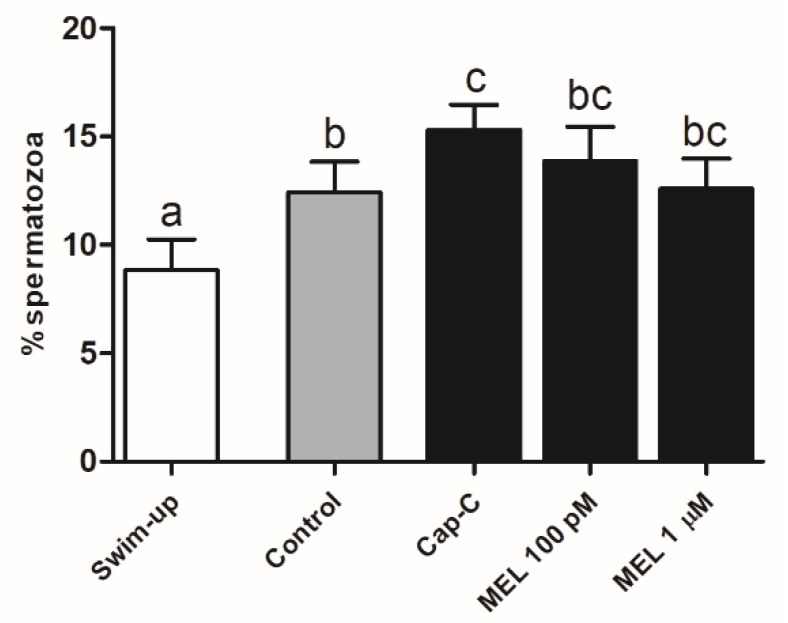
Percentage of live sperm with high nitric oxide levels (PI-/DAF+) assessed by flow cytometry before (swim-up, white bars) and after in vitro capacitation without (control, grey bars) and with cAMP-elevating agents (Cap-C, black bars) and with L-arginine (L-arg, 10 mM) or L-NAME (100 µM or 1 mM). Data are shown as mean ± S.E.M (*n* = 7). Different letters indicate significant differences (*p* < 0.05).

The presence of melatonin did not significantly modify the percentage of live spermatozoa with high nitric oxide levels (PI-/DAF+) compared to the capacitated control sample (Cap-C), although the values obtained were similar to the control sample without cAMP-elevating agents (Figure 9). 

## 3. Discussion

The role of nitric oxide in sperm physiology has been widely investigated. It affects sperm motility [1,3], capacitation [5,6] and the acrosome reaction [8]; acts as a chemoattractant and enhances zona pellucida-binding [10]. These actions are regulated by the active nitric oxide synthase (NOS), which generates nitric oxide from L-arginine. 

In the present study, we have demonstrated, for the first time, the presence of the three isoforms of NOS in ram spermatozoa and their capacity to produce nitric oxide. Constitutive isoforms, neural (nNOS) and endothelial (eNOS), were detected by Western blotting but not the inducible form (iNOS). Both nNOS and eNOS had previously been identified in spermatozoa from other species, such as human, mouse, bull, boar, stallion and cat, but the inducible form (iNOS) was only detected in some of them (reviewed by [36]). At least in somatic cells, the iNOS should not be expressed without the presence of a stimulus such as inflammation, lipopolysaccharides or cytokines [35,37,38,39]. If iNOS is present in low quantities under normal conditions, this would explain why it was not possible to detect it by Western blotting in the current work. Moreover, although eNOS and nNOS were evidenced by Western blot, densitometric quantification did not reveal any significant change in their levels after in vitro capacitation. Other techniques to measure NOS activity should be addressed in future studies. 

However, the three NOS isoforms were detected by IFI, all being located mainly in the sperm head, with similar labeling patterns. We described two immunotypes for each isoform (immunotypes 1 and 2 for eNOS and immunotypes 3 and 4 for nNOS and iNOS) related to the presence or absence of fluorescence in the apical edge. Meiser and Schulz [20] localized the nNOS isoform in the apical region of the head and tail of bull spermatozoa, whereas the eNOS was located only in the apical region. In ejaculated boar spermatozoa, eNOS and nNOS were identified in the acrosome and iNOS in the whole head, neck and flagellum [40]. However, no immunotypes or changes in NOS localization after in vitro capacitation were reported for bull or boar spermatozoa. In the current work, capacitation induced by the addition of cAMP-elevating agents led to a decrease in the percentage of ram spermatozoa showing labeling on the apical edge for all three isoforms. This would be in concordance with the results reported for mice spermatozoa [22], where NOS disappeared off the head when spermatozoa were incubated under capacitating conditions, although the NOS isoforms were not identified in that work. Thus, these findings suggest that nitric oxide synthase may change its localization in the sperm cell so as to modulate its activity and, consequently, the nitric oxide production in ram spermatozoa. 

After demonstrating the presence of NOS in ram spermatozoa and its distribution changes after in vitro capacitation, we investigated how its product, nitric oxide, fluctuates during incubation in capacitating conditions. Our results showed a significant increase in NO· levels, which has also been reported for other species such as human [8] and boar [41]. The addition of L-arginine raised NO· sharply, as expected for a NO· precursor. This extra amount of NO· led to a major percentage of acrosome-reacted sperm, as described for other species [19,42,43,44].

Ambiguous results have been reported about the role of NO· in sperm motility. Some studies claimed the beneficial effect of NO· in sperm motility [1,45], while others concluded that the induction of NO·, for example by L-arg, was detrimental to progressive motility [21,46,47,48]. In our study, the increment in NO· induced by the addition of L-arginine had no effect on sperm motility, probably due to how the samples were capacitated and, therefore, hyperactivated, which could mask the putative effect of L-arginine.

Incubation with a NOS inhibitor, L-NAME, was able to reduce the nitric oxide levels only in ram sperm samples incubated with cAMP-elevating agents, but this slight decrease did not provoke changes in sperm motility or the capacitation state. Reported effects of L-NAME on sperm from different species are contradictory. Whereas some authors did not observe any action of this compound [5,49], others clearly found effects on sperm physiology [5,7,43,44,49,50]. These conflicting results could be due to the different responses of spermatozoa from different species to the L-NAME and also the varied protocols used to evaluate its actions. In any case, the small decrease in NO· levels that L-NAME induced in ram spermatozoa was not able to inhibit capacitation or the acrosome reaction, probably because the process had already been triggered. 

Melatonin is a well-known antioxidant that also plays an important role in sperm functionality [30,51], especially in ram spermatozoa [24,25]. Our group have previously reported the bimodal action of melatonin on ram sperm in in vitro capacitation, as well as its protective role against apoptosis and oxidative stress [27,52]. The results in this study supported our previous findings, confirming that melatonin at micromolar concentrations prevents sperm capacitation, whereas at picomolar concentrations, it increases the percentage of acrosome-reacted, and decreases noncapacitated, spermatozoa. We further investigated whether this melatonin effect was mediated by its interaction with NOS and/or nitric oxide metabolism. Our results showed that melatonin was able to induce changes in NOS distribution in capacitated ram sperm. Thus, the presence of melatonin prevented changes in nNOS immunotypes during capacitation with high-cAMP agents. We might postulate that the nNOS moves out of the acrosomal region due to the membrane reorganization, which occurs during the capacitation process, and that melatonin modifies the nNOS location in the same way that it modulates ram sperm capacitation. On the other hand, melatonin enhanced the effect of capacitation with high-cAMP agents on the iNOS distribution, further decreasing the apical-edge labeling. This behavior could be explained by the different roles of the NOS isoforms. Staicu et al. [40] postulated that the constitutive isoforms (eNOS and nNOS) could be involved in sperm capacitation and that iNOS could be related to inflammation or stress, so the changes observed in iNOS during in vitro capacitation with melatonin could not exactly be related to this process. Taking into account the bimodal role that melatonin has on ram sperm in in vitro capacitation, stimulating this process at low concentrations (100 pM) and preventing it at high doses (1 µM) [27], it is quite surprising that both melatonin concentrations have similar effects on iNOS and nNOS distribution. Finally, no changes in eNOS location were detected after capacitation in high-cAMP conditions or with the presence of melatonin. Likewise, no significant changes were observed in the levels of these enzymes analyzed by Western blot after densitometric quantification. To further confirm these results, enzymatic assays should be carried out in future studies. 

Finally, the addition of melatonin to the high-cAMP capacitated samples did not affect the nitric oxide production, as L-arginine or L-NAME did. Therefore, we could postulate that the action of melatonin on ram sperm capacitation is not mediated through the NO· pathway. Du Plessis et al. [28] described a scavenging effect of melatonin in human spermatozoa after the evaluation of DAF2-DA fluorescence, but, contrary to our previous publications [52], they did not see any effect on the reactive oxygen species (ROS). This might be due to species-specific differences or, more likely, to the fact that they used raw semen, and we studied the melatonin effects in capacitated samples. Nonetheless, it could still be postulated that there is some effect of melatonin on nitric oxide production, because the percentage of spermatozoa with high NO· levels in samples capacitated with melatonin went down to values similar to those in the control sample (without cAMP-elevating agents). 

Considering the results obtained from this work taken together, we have evidenced, for the first time, the presence of the nitric oxide synthase isoforms in ram spermatozoa. Moreover, we have proved that nitric oxide plays a role in ram sperm capacitation, given that the location of some NOS isoforms changed and the levels of NO·increased after in vitro capacitation. Furthermore, the induction of NO· synthesis by the addition of L-arginine caused an increment in the percentage of acrosome-reacted spermatozoa. Melatonin modified the location of NOS isoforms but not their protein expression profile or the NO· levels. Thus, the effect produced by this hormone on ram sperm capacitation does not seem to take place through the nitric oxide pathway.

## 4. Materials and Methods 

### 4.1. Semen Collection and Processing

Semen was collected from nine *Rasa Aragonesa* rams (2–4 years old) using an artificial vagina. All the rams belonged to the National Association of *Rasa Aragonesa* Breeding (ANGRA) and were kept at the Experimental Farm of the University of Zaragoza Veterinary School under uniform nutritional conditions. After a period of two days’ abstinence, two successive ejaculates were collected, and second ejaculates were pooled and processed together to avoid individual differences [53]. Ejaculates were kept at 37° until analysis. 

All experimental procedures were performed under the project license PI34/11 approved by the University of Zaragoza Ethics Committee for Animal Experiments.

A seminal plasma-free sperm population was obtained using a dextran swim-up procedure [54] performed in a medium with the following composition: 200 mM sucrose, 50 mM NaCl, 18.6 mM sodium lactate, 21 mM HEPES, 10 mM KCl, 2.8 mM glucose, 0.4 mM MgSO_4_, 0.3 mM sodium pyruvate, 0.3 mM K_2_HPO_4_, 5 mg/mL bovine serum albumin (BSA), 30 mg/mL dextran, 1.5 IU/mL penicillin and 1.5 mg/mL streptomycin, pH 6.5.

The sperm concentration was calculated in duplicate using a Neubauer chamber (Marienfeld, Lauda-Königshofen, Germany). Initial concentration of spermatozoa in the collected ejaculates was 3–4 × 10^9^ cells/mL and 3–4 × 10^8^ cells/mL after the swim-up procedure.

### 4.2. In Vitro Capacitation

Swim-up-selected spermatozoa, in aliquots of 1.6 × 10^8^ cells/mL, were incubated for 3 h at 39 °C in a humidified incubator with 5% CO_2_ in the air. Incubations were performed in a complete TALP medium [55] containing 100 mM NaCl, 3.1 mM KCl, 25 mM NaHCO_3_, 0.3 mM NaH_2_PO_4_, 21.6 mM Na lactate, 3 mM CaCl_2_, 0.4 mM MgCl_2_, 10 mM HEPES, 1 mM Na pyruvate, 5 mM glucose and 5 mg/mL bovine serum albumin (BSA), with a pH of 7.2. A high-cAMP medium already proven for capacitating ram spermatozoa [56,57] composed of 1 mM dibutyryl (db)-cAMP, 1 mM caffeine, 1 mM theophylline, 0.2 mM okadaic acid and 2.5 mM methyl-b-cyclodextrin was added to the TALP medium to induce in vitro capacitation (capacitated control sample). Another control sample incubated in a TALP medium alone without the addition of cAMP-elevating agents was maintainted under the same incubation conditions (control sample).

To further investigate the role of nitric oxide during in vitro capacitation, NG-nitro-L-arginine methyl ester (L-NAME), a nonselective nitric oxide synthase inhibitor, was added to the TALP (control) and high-cAMP samples at final concentrations of 100 µM and 1 mM, whereas L-arginine (L-arg), a NOS substrate, was used in both media at a 10 mM concentration, based on the previous work of Hassanpour et al. [46]. 

Melatonin was solubilized in dimethyl sulphoxide (DMSO) and phosphate-buffered saline (PBS, 137 mM NaCl, 2.7 mM KCl, 8.1 mM Na_2_HPO_4_ and 1.76 KH_2_PO_4_, pH 7.4) and added to the high-cAMP capacitated samples at a final concentration of 1 µM or 100 pM. The final concentration of DMSO in all the melatonin samples was 0.1%. The same DMSO concentration was included in the control samples incubated in capacitating conditions. 

The experimental groups were defined as follows: swim-up, for the spermatozoa selected by the dextran swim-up method before inducing in vitro capacitation, control, for the spermatozoa incubated under capacitating conditions (3 h at 39 °C in 100% humidity and 5% CO_2_ in the air) in TALP medium and capacitated control (Cap-C), for spermatozoa incubated under capacitating conditions in TALP medium with the cAMP-elevating agents. The concentrations of each treatment (L-arg, L-NAME or melatonin) added to each condition were indicated when necessary.

### 4.3. Sperm Motility Evaluation

Motility kinematic parameters, total motility and progressive motility were evaluated using a computer-assisted sperm analysis (CASA) system, the OpenCASA motility module, a free and open-source software that we recently developed (Alquezar-Baeta et al., 2019). Two drops of 2 µL of each sample, diluted to a final concentration of 3 × 10^7^ cells/mL, were placed in a Makler counting chamber (Sefi-Medical Instruments, Haifa, Israel) prewarmed and maintained at 37 °C during all the analyses by a heated slide holder. The spermatozoa were recorded with a video camera (Basler acA1920; Basler Vision Components, Ahrensburg, Germany) mounted on a microscope (Nikon Eclipse 50i, Nikon Instruments Int, Tokyo, Japan) equipped with a 10× negative-phase contrast lens.

Recorded videos were evaluated with the following settings: 60 frames per second, 120 frames, 800 × 600 pixel image resolution, 10 µm^2^ minimum cell size, 100 µm^2^ maximum cell size, progressive motility, STR (straightness coefficient) >80% and VAP (mean velocity)>90%, 10 µm/s minimum VCL (curvilinear velocity), 100 µm/s VCL lower threshold, 200 µm/s VCL upper threshold, 30 frames minimum track length and 20 µm maximum displacement between frames.

### 4.4. Western Blotting

For NOS detection, sperm proteins were extracted from swim-up-selected and capacitated spermatozoa, as previously described by Colas et al. [56]. Semen was diluted in PBS (1.6 × 10^8^ cells/mL) and centrifuged at 900× *g* for 5 min. The supernatant was discarded, and the pellet was resuspended in 200 µL of extraction sample buffer (ESB; 125 mM Tris–HCl (pH 6.8) and 2% SDS (w/v)) with a 10% protease inhibitor’s cocktail (Sigma-Aldrich Corporation, St. Louis, MO, USA). After incubation at 100 °C in a sand bath for 5 min, samples were centrifuged again at 7500× *g* for 5 min at 4 °C. The supernatant was recovered, and β-mercaptoethanol, glycerol and bromophenol blue (in 10 % glycerol) were added to final concentrations of 5%, 1% and 0.002 % (v/v), respectively. Lysates were stored at −20 °C.

Sperm extracted proteins (40 µL) were separated in one dimension on 10% sodium dodecyl sulphate polyacrylamide gel electrophoresis (SDS-PAGE) [58] and transferred during 20 h at 22 V onto a polyvinylidene difluoride (PVDF) membrane (Immobilon-P, Millipore, Bedford, MA, USA) using a wet transfer unit (Mini Trans-Blot Electrophoretic Transfer Cell, Bio-Rad, Hercules, CA, USA). Nonspecific sites on the PVDF membrane were blocked with 5% BSA (*v*/*v*) in PBS for 4 h. The proteins were immunodetected by incubating with rabbit primary anti-eNOS (Abcam Cat# ab5589, RRID: AB_304967), anti-nNOS (Abcam Cat# ab76067, RRID: AB_2152469) or anti-iNOS (Abcam Cat# ab3523, RRID: AB_303872) antibodies, whereas a mouse anti-tubulin antibody (Santa Cruz Biotechnology Cat# sc-8035, RRID: AB_628408) was used as a loading control. The anti-nNOS, anti-iNOS and anti-eNOS antibodies were diluted 1/500, and the anti-tubulin antibody was diluted 1/1000 in 0.1% (v/v) Tween-20 in PBS containing 1% (*w*/*v*) BSA overnight at 4 °C. To check the specificity of the antibodies, the amino acid sequences from ovine eNOS, iNOS and nNOS, obtained from UniProt Knowledgebase (UniProtKB, at https://www.uniprot.org), were aligned (Clustal Omega, Multiple Sequence Alignment program) with the peptide sequences used by the manufacturer to raise the anti-NOS antibodies (when available) or with the human NOS sequences. The high homology found between the sequencies (93.75% for eNOS, 91.86% for nNOS and 85.70% for iNOS) suggests that these antibodies can be suitable for their use in ovine. We used the commercial cell lysates recommended explicitly by the antibody manufacturer as positives controls: rat lung extract for eNOS, mouse brain extract for nNOS (both from Santa Cruz Biotechnology, Inc., Dallas, TX, USA) and mouse macrophage activated with IFNɣ/LPS lysate for iNOS (BD Biosciences, San Jose, CA, USA). After extensive washing with 0.1% (v/v) Tween-20 PBS, membranes were incubated for 75 min at room temperature with the secondary antibodies donkey anti-rabbit (IRDye 680RD donkey anti-rabbit IgG antibody, LI-COR Biosciences Cat# 926-68073, RRID: AB_10954442) and donkey anti-mouse (RDye 800CW donkey anti-mouse IgG antibody, LI-COR Biosciences Cat# 926-32212, RRID: AB_621847) diluted 1/15,000 in 0.1% (v/v) Tween-20 PBS containing 1% (v/v) BSA. After extensive washing, the membranes were scanned using the OdisseyCLx Imaging System (LI-COR Biosciences, Lincoln, NE, USA). 

Western blot images were quantified using Odyssey Clx Infrared Imaging System software (Li-COR Biosciences, Lincoln, NE, USA) to determine the relative intensity of the NOS protein bands normalized to the tubulin control.

### 4.5. Indirect Immunofluorescence

The localization of the three isoforms of NOS in ram spermatozoa was investigated by indirect immunofluorescence analyses. Sperm samples were diluted (8 × 10^6^ cells/mL) in PBS and fixed in 0.5% formaldehyde at room temperature for 20 min. After that, cells were centrifugated at 900× *g*, and the pellet was resuspended in 500-µL PBS. Forty microliters of cell suspension were placed onto Superfrost slides (Superfrost Plus; Thermo Scientific, Waltham, MA, USA) and permeabilized with 0.5% (*v/v*) Triton X-100 in PBS for 15 min. The cells were then fixed again with paraformaldehyde 1.25% in Tris-HCl 0.5 M for 5 min and washed three times with PBS; after which, the nonspecific binding sites were blocked with 5% (*w/v*) BSA in PBS for 5 h in a wet chamber. The slides were rewashed in PBS and incubated at 4 °C overnight in a wet chamber with the primary antibody anti-eNOS (Abcam Cat# ab5589, RRID: AB_304967), anti-nNOS (Abcam Cat# ab76067, RRID: AB_2152469) or anti-iNOS (Abcam Cat# ab3523, RRID: AB_303872), all diluted 1/25 in PBS with 1% (*v/v*) BSA. As indicated for the Western blotting experiments (4.4), the specifity of these antibodies was reviewed by multiple aminoacid sequence alignment. Next morning, samples were washed three times with PBS and incubated with the secondary antibody (Alexa Fluor 488 chicken anti-rabbit; Thermo Fisher Scientific; Cat#A-21441, RRID: AB_2535859) and diluted 1/800 (*v/v*) in PBS with 1% (*v/v*) BSA for 90 min at room temperature in a wet chamber. The slides were then washed three times with PBS before the addition of 6 μL of 0.22 M 1,4-Diazabicyclo[2.2.2]octane (DABCO, Sigma-Aldrich Corporation, St. Louis, MO, USA) in glycerol:PBS (9:1 *v/v*) to enhance and preserve cell fluorescence. The slides were then covered with a coverslip and sealed with transparent enamel. Cells were visualized with a Nikon Eclipse E400 microscope (Nikon, Tokyo, Japan) under epifluorescence illumination using a B-2A filter (×1000). All samples were processed in duplicate, and at least 150 spermatozoa were scored per slide.

### 4.6. Flow Cytometry Analysis

All the measurements were performed on a Beckman Coulter FC 500 flow cytometer (Beckman Coulter Inc., Brea, CA, USA) equipped with two excitation lasers (air-cooled Argon ion laser 488 nm and Red Solid state laser 633 nm); five absorbance filters (FL1-525, FL2-575, FL3-610, FL4-675 and FL5-755; ±5 nm each band pass filter) and CXP software. A minimum of 20,000 events were evaluated in all the experiments. The sperm population was identified for further analysis on the basis of its specific forward (FS) and side-scatter (SS) properties; thus, other events were excluded. A flow rate stabilized at 200–300 cells/s was used.

#### 4.6.1. Evaluation of Sperm Membrane Integrity

Sperm viability, considered as the integrity of the cell plasma membrane, was assessed by adding 3 µL of 1 mM carboxyfluorescein diacetate (CFDA; Sigma-Aldrich Corp., St. Louis, MO, USA), 1.5 mM propidium iodide (PI; Sigma-Aldrich Corp., St. Louis, MO, USA) and 5 µL of formaldehyde (0.5% (v/v) in water) to a final concentration of 5 × 10^6^ cells/mL in a 300-µL volume [59]. Samples were incubated at 37 °C in darkness for 15 min. For the sperm viability analysis, the Argon laser and filters FL1-525 and FL4-675 nm were used to avoid overlapping. The monitored parameters were FS log, SS log, FL1 log (CFDA) and FL4 log (PI). The percentage of viable spermatozoa (CFDA+/PI-) was evaluated.

#### 4.6.2. Intracellular Nitric Oxide Levels

Intracellular levels of nitric oxide (NO·) were determined using 4,5-diaminofluorescein diacetate (DAF-2; Cayman Chemical, Ann Arbor, MI, USA). DAF-2 is a cell-permeable probe that in the presence of NO· is metabolized to the highly fluorescent triazolofluorescein (DAF-2T) [60]. Samples of 5 × 10^6^ cells/mL were stained with 10 µM DAF-2 and 1.5 mM PI, incubated at 37 °C in darkness for 15 min and analyzed by flow cytometry using the argon laser and filters FL1-525 and FL4-675 nm. The monitored parameters were FS log, SS log, FL1 log (DAF-2) and FL4 log (PI). Three populations were distinguished: viable cells with high NO· production (PI-/DAF-2+), viable cells with low NO· production (PI-/DAF2-) and nonviable cells with low NO· production (PI+/DAF).

### 4.7. Determination of Capacitation Status

Capacitation status was determined by chlortetracycline (CTC) staining, a fluorescence assay for the evaluation of capacitation and acrosome reaction-like changes that we previously validated in ram sperm [57]. A CTC solution (750 µM; Sigma-Aldrich Corp., St. Louis, MO, USA) was prepared daily in a buffer containing 20 mM Tris, 130 mM NaCl and 5 µM cysteine, pH 7.8, and passed through a 0.22-µm filter (Merck Millipore, Darmstadt, Germany). After that, 20 µL of CTC solution was added to 18 µL of sperm sample, fixed with 5 µL of 1.25% (*w/v*) paraformaldehyde in 0.5 M Tris-HCl (pH 7.8) and incubated at 4 °C in the dark for 30 min. Six microliters of the stained sample was placed onto a glass slide and mixed with 2 µL of 0.22 M triethylenediamine (DABCO; Sigma-Aldrich Corp., St. Louis, MO, USA) in glycerol:PBS (9:1 v/v). The samples were covered with 24 × 60-mm coverslips, sealed with transparent enamel and stored in the dark at −20 °C until evaluation. The samples were examined using a Nikon Eclipse E-400 microscope (Nikon Corporation, Kanagawa, Japan) under epifluorescence illumination with a V-2A filter, and at least 200 spermatozoa were scored per sample. Three sperm patterns were identified [61]: noncapacitated (even distribution of fluorescence on the head, with or without a bright equatorial band), capacitated (with fluorescence in the acrosome) and acrosome-reacted cells (showing no fluorescence on the head, with or without a bright equatorial band).

### 4.8. Statistical Analysis

Differences between the groups in motility, nitric oxide levels, viability, CTC staining and NOS immunotypes were analyzed by means of the chi-square test. Differences in NOS expression profiles evaluated by Western blot were analyzed by ANOVA, followed by the Bonferroni post hoc test after evaluation of the data distribution by the Kolmogorov–Smirnov test. All statistical analyses were performed using GraphPad Prism 5 (v. 5.03; GraphPad Software, La Jolla, CA, USA).

## Appendix A

Sperm viability, evaluated by CFDA/PI staining and assessed by flow cytometry. No significant differences between groups were found.
